# In memoriam - Affonso Berardinelli Tarantino

**DOI:** 10.1590/S1806-37132014000500001

**Published:** 2014

**Authors:** Jorge Ibrain de Figueira Salluh

**Affiliations:** Graduate Program, Brazilian National Cancer Institute, Rio de Janeiro, Brazil

**Figure:**
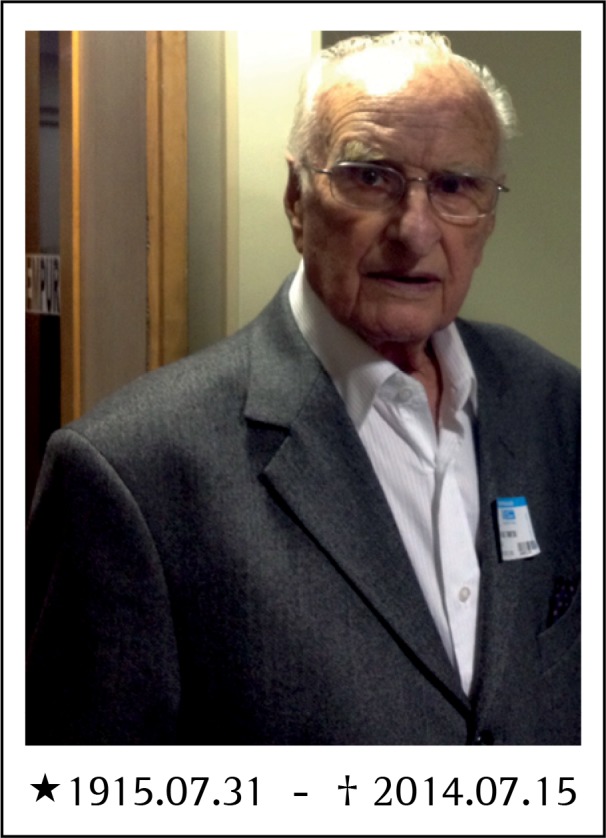

"*Il padre é il maestro, e colui che plasma la nostra mente*" ("The father is the master, and the one who shapes our mind"). Those words, unpretentiously displayed on the pulmonology classroom wall in the school of medicine and yet clearly visible, left no room for doubt. More than simply learning pulmonology, students there would have the opportunity to immerse themselves in culture and humanity. 

Affonso Berardinelli Tarantino was the "master" of many, not only because he was a classic Professor (with a capital P) or because he clearly had a natural talent for teaching (combined with vast medical knowledge) but also (and especially) because he was able to impart his knowledge in a simple and efficient manner. Therefore, all of those who, however briefly, enjoyed his company, were his students. 

Born in 1915 in the city of São José dos Campos, in the Paraíba Valley, Brazil, Tarantino lived for nearly a century, in which he acquired and disseminated knowledge of pulmonology and phthisiology. In the first half of the last century, Tarantino furthered his training at the prestigious Forlanini Institute, in Italy, which was on the cutting edge of phthisiology with the methods developed by Monaldi and Carlo Forlanini. Back in Brazil, Tarantino devoted himself to the teaching and clinical practice of pulmonology in the city of Rio de Janeiro, Brazil. However, it was essentially through books that he was able to pour his immense knowledge over the past four decades. The now classic "Doenças Pulmonares"^(^ 1 ^)^ was a faithful mirror of its primary author and editor. It brought together a team of star pulmonologists from various Brazilian states (all of whom were dear friends of Tarantino's) and had the striking characteristic of being thorough and profound without ever abandoning a practical discourse. Tarantino used to say that he had to be able to communicate with physicians anywhere in Brazil, even in the most remote parts of the country. His book (which is currently in its sixth edition) was thus written and can be found in major university libraries, study rooms, and physician offices nationwide. It is a ubiquitous reference for scholars of diseases of the respiratory system. 

Tarantino was the coauthor and editor of several medical books, on which he left his mark. Although he was essentially a phthisiologist, he was also a scholar of pneumonia and sarcoidosis, the latter being the theme of his tenure thesis. Tarantino also rewarded us with his reflections and his privileged and generous view of the world in books such as "Repetrechos",^(^ 2 ^)^ in which each page allows those with a keen eye to gain further understanding of the magnificent teacher and human being that he was. 

Therefore, although I feel it is my duty to write a eulogy, I feel unable to write one that is worthy of the man and professor whose company I had the immense privilege of enjoying for over twenty years. My mentor and my godfather, Tarantino was the one who sent me on the path to becoming a pulmonologist. 

Over the course of nearly a hundred years, Tarantino was a modern man; he lived his life unfettered by the notion that time is fleeting and had more than simple students: he had disciples. We are all diminished by his passing last July. His lessons, books, and history will remain with us. Because opera was one of Tarantino's greatest passions, I will leave you with a quote that might shed some light on life and its tortuous pathways. The quote is from *Recondita Armonia*, the first aria in Giacomo Puccini's Tosca: "*L'arte nel suo mistero le diverse bellezze insiem confond*" (Art has a mysterious way of blending the contrasting beauties). Such contrasting beauty can be found between our memories of Tarantino and in our sadness over losing him.
