# Supplementary material for: In memoriam - Affonso Berardinelli Tarantino
Source: J Bras Pneumol. 2014 Sep-Oct;40(5):453–4. doi: 10.1590/S1806-37132014000500001 (PMC4263326; doi:10.1590/S1806-37132014000500001)
Supplement: Translation

# Translation: In memoriam - Affonso Berardinelli Tarantino

**Authors:** Jorge Ibrain de Figueira Salluh

**Affiliations:** Programa de Pós-Graduação, Instituto Nacional do Câncer, Rio de Janeiro, RJ, Brasil

"*Il padre é il maestro, e colui che plasma la nostra mente*". Dispostas de modo despretensioso e, ao mesmo tempo, amplamente visíveis, essas palavras escritas na parede da sala de aula do curso de pneumologia da faculdade de medicina não deixavam margem a dúvidas. Mais do que pneumologia, os alunos teriam a oportunidade de estar imersos em aulas onde se respiravam cultura e humanidade. 

Affonso Berardinelli Tarantino foi o mestre de muitos, não somente porque foi um clássico Professor (com maiúscula) e porque tinha clara vocação natural para a docência, aliados a uma imensa cultura médica. Mas especialmente porque toda sua erudição fluía com uma comunicação simples e eficiente. Assim, foram seus alunos todos aqueles que conviveram com ele, mesmo se por breves períodos.

Nascido em São José dos Campos, no Vale do Paraíba, em 1915, Affonso Tarantino atravessou quase um século absorvendo e difundindo o conhecimento em pneumologia e tisiologia. Tarantino, ainda na primeira metade do século passado, complementou a sua formação no prestigioso Instituto Forlanini na Itália, onde se estudava e se praticava a fronteira do conhecimento em tisiologia com os métodos então desenvolvidos por Monaldi e Carlo Forlanini. De volta ao Brasil, Affonso Tarantino dedicou-se à docência e à clínica na cidade do Rio de janeiro, mas foi essencialmente através dos livros que, nas últimas quatro décadas, pode desaguar seu imenso conhecimento. O hoje clássico "Doenças Pulmonares"^(^ 1 ^)^ era um espelho fiel do seu autor maior e editor. Reunia um time de estrelas da pneumologia de diversos estados brasileiros (queridos amigos do mestre) e tinha como característica marcante ser minucioso e profundo sem nunca se dissociar de um discurso prático. Tarantino gostava de dizer que tinha que poder sempre falar ao médico do interior do país. E assim construiu seis edições onipresentes nas bibliotecas das grandes universidades, nas salas de estudos de alunos e nos consultórios por todo país. Referências ubíquas para os estudiosos de doenças do sistema respiratório.

Tarantino colaborou e editou diversos livros médicos nos quais imprimiu a sua marca. Foi essencialmente um tisiologista, mas foi grande estudioso das pneumonias e também da sarcoidose, essa última tema de sua livre-docência. Pôde ainda nos brindar com suas reflexões e sua visão privilegiada e generosa do mundo em livros como "Repetrechos",^(^ 2 ^)^ onde cada página permite, a um olhar atento, decifrar um pouco mais do mestre e do ser humano magnífico que era.

Por isso tudo, sinto-me ao mesmo tempo incumbido desta missão, mas incapaz de fazer um necrológio a altura desse homem e professor com quem tive o imenso privilégio de conviver por mais de vinte anos. Meu mestre, meu padrinho e que me fez trilhar por esta especialidade.

Certo é que em seu século de vida, Affonso Tarantino foi um homem moderno e desconheceu os limites do tempo, teve mais discípulos do que simplesmente alunos, e, com sua partida nesse mês de julho, ficamos todos mais pobres. Entre nós ficam as lições, os livros e a história. Fica também uma reflexão que, de alguma forma, nos ajuda a explicar a vida e seus caminhos tortuosos. Essa vem de uma das maiores paixões de Affonso Tarantino: a ópera. Assim, nas palavras da "Recondita Armonia" da Tosca de Puccini: "*L'arte nel suo mistero le diverse bellezze insiem confond*" (A arte no seu mistério combina entre si as diferentes belezas). Beleza que também se manifesta, de alguma forma, nas lembranças mescladas às imensas saudades que deixa.
